# Significance of Serum Adiponectin and Insulin Resistance Levels in Diagnosis of Egyptian Patients with Chronic Liver Disease and HCC

**DOI:** 10.31557/APJCP.2019.20.6.1833

**Published:** 2019

**Authors:** Hend A Radwan, Ehab H Elsayed, Omneya M Saleh

**Affiliations:** *Department of Internal Medicine, National Research Center, Cairo, Egypt. *

**Keywords:** Hepatocellular carcinoma (HCC), Chronic liver disease (CLD), Adiponectin, Insulin Resistance (IR)

## Abstract

One possible hypothesis for pathogenesis of hepatocellular carcinoma is deregulated expressed adipokines (adipose tissue cytokines). Chronic inflammation in the cirrhotic liver adipose tissue is associated with a modification in adipokine secretion. Changes in serum levels of adiponectin are known to be associated with the development of insulin resistance. Increased insulin resistance is a pathophysiological feature of nonalcoholic fatty liver disease (NAFLD), one of the most common causes of chronic liver disease. In addition, it was suggested that liver cancer development is probably connected with insulin resistance. The aim of this study is to evaluate the significance of serum Adiponectin level and insulin resistance in patients with chronic liver disease and hepatocellular carcinoma.

**Patient and Methods:**

100 patients were enrolled in this cross sectional study and divided as following: Group I: 52 HCV patients with chronic liver disease (CLD).Group II: 48 patients with hepatocellular carcinoma (HCC). For all subjects, Serum Adiponectin and Insulin Resistance parameters (Fasting serum Insulin, Fasting serum Glucose, HOMA IR) were measured.

**Results:**

Serum Adiponectin was significantly lower in patients with hepatocellular carcinoma (p=0.000 ) and it is inversely correlated to tumor size and the number (p= 0.0001).Meanwhile, Insulin Resistance parameters (Fasting s. Insulin, Fasting s. Glucose, HOMA IR) were significantly higher in HCC patients than CLD patients (p= 0.0001).

**Conclusion:**

Insulin Resistance is significantly associated with the development of HCC. Serum level of Adiponectin may guard against HCC development among patients with chronic liver disease.

## Introduction

Adiponectin, a protein normally produced by the adipose tissue, is considered a potent modulator of lipid and glucose metabolism (Ye and Scherer, 2013).

It circulates at high concentrations (0.5–30 μg/ml) in plasma under normal physiological conditions. Adiponectin levels are hormonally regulated: the hormone testosterone inhibits adiponectin secretion, triggering lower levels of adiponectin in men (Wang et al., 2008).

This protein modulates hepatic fibrogenesis and affects various biological processes that are involved in liver function, including angiogenesis, vasodilation, inflammation and deposition of extracellular matrix proteins (Buechler et al., 2017).

In the liver, adiponectin increases fatty acid β-oxidation, thereby decreasing hepatic TG [triacylglycerol (triglyceride)] content and hepatic insulin resistance. Adiponectin-associated metabolic effects are exerted via corresponding receptors, AdipoR1 (adiponectin receptor 1) and AdipoR2 (adiponectin receptor 2). Although AdipoR1 is considered important for mediating adiponectin effects in skeletal muscle, AdipoR2 appears to be the predominant player in liver (Kadowaki et al., 2008; Lemoine et al., 2009).

Accumulating epidemiologic evidence suggests that excess body weight is an independent risk factor for primary liver cancer (Saunders et al., 2010). Even in Japan, where the prevalence of overweight and obesity is lower than that in Western countries and the main cause of liver cancer is chronic hepatitis C virus (HCV) infection, overweight or obesity seem to increase the risk of liver cancer (Tanaka et al.,2012). The causal association between excess body weight and liver cancer development is probably connected with insulin resistance (Bugianesi et al., 2005, Inoue et al., 2012). However, the underlying mechanism by which excess body weight promotes liver cancer development is still not fully understood.

Insulin resistance (IR) is a systemic disease affecting the nervous system, muscles, pancreas, kidney, heart, and immune system, in addition to the liver. It is a common characteristic of the metabolic syndrome and its related features (Bugianesi et al., 2005). NASH (non- alcoholic steatohepatitis) is the liver manifestation of metabolic syndrome, which includes insulin resistance, obesity and dyslipidemia (Yu et al., 2013).

In NAFLD (non- alcoholic fatty liver disease), it was suggested that there is a complex interaction between genes and the environment that favors or enhances IR and the phenotypic expression of NAFLD in individual patients (Bugianesi et al., 2005).

Insulin resistance was found to parallel the liver fibrosis stage (Petta et al., 2011). So, it is important to understand the pathogenesis of insulin resistance in patients with chronic hepatitis C, as insulin resistance seems to be involved in the disease progression and success of treatment (Kawaguchi et al., 2011).

The onset of a proinflammatory state is provoked by several factors secreted or expressed in the adipocyte, which may be limited to the liver or more extensively expressed throughout the body (Bugianesi et al., 2005).The development of insulin resistanceis known to be associated withchanges in serum levels of adiponectin, leptin, tumor necrosis factor-alpha and interleukin-6 (Polyzos et al., 2009).

Earlier experimental studies suggested that adiponectin played a protective role in carcinogenesis via insulin sensitization, antiproliferation, anti-inflammation, and angiogenesis regulation (Barb et al., 2007) and data supported epidemiologic evidence that adiponectin levels were inversely associated with the risk of obesity-related malignancies, such as breast, colorectal, endometrium, and prostate cancers (Barb et al., 2007). These results also suggested that elevated levels of adiponectin would be associated with a reduced risk of primary liver cancer linked with obesity, and that hyperadiponectinemia might suppress liver tumorigenesis (Wieser et al., 2012). Indeed, experimental studies indicated that adiponectin treatment increased apoptosis of hepatocelluar carcinoma, the most common form of primary liver cancer, and inhibited its proliferation (Man et al., 2010; Saxenaet al., 2010). However, it has been pointed out that hyperadiponectinemia reflects the progression of liver disease leading to the development of liver cancer, as the liver is the main organ of adiponectin metabolism (Wieser et al., 2012).

In the current study, we aim to evaluate the significance of serum Adiponectin level and insulin resistance among patients with chronic liver disease and hepatocellular carcinoma and to explore the relation of Adiponectin and insulin resistance to the metabolic profile in these patients.

## Materials and Methods

This study is cross sectional comparative type.100 patients were recruited from the outpatient clinic of Eldemerdash Hospital, Ain Shams university Hospitals.

*Patients*

*They were divided into two groups*

Group I: 52 HCV infected patients with chronic liver disease (CLD) without any evidence of hepatic focal lesions as excluded by ultrasonography and AFP. Diagnosis of chronic liver disease was based on standard clinical, biochemical, ultrasonographic criteria, and pathological data whenever feasible. This group served as a diseased control group. Exclusion criteria of group I:-Presence of any hepatic focal lesion (HFL). -Presence of any other malignancy.

Group II: 48 patients with hepatocellular carcinoma (HCC) on background of liver disease. The diagnosis of HCC was established by imaging + AFP ± histopathology according to AASLD practice guidelines (Bruix and Sherman, 2005). Exclusion criteria of group II:-Presence of any other malignancy and/or Presence of metastasis.

*Methods*

All patients were subjected to the following: A written consent and oral explanation of the whole procedure. Clinical assessment including: Careful history taking, proper physical examination and calculation of Body Mass Index (BMI= weight in kilograms / height in meters²). Laboratory investigations including: Complete blood picture, renal function tests (Serum creatinine and BUN, liver profile (Alt, AsT, serum Albumin, total and direct bilirubin and alpha – fetoprotein), biomarkers for hepatitis B virus (HBsAg, anti-HBcAb”total”) and hepatitis C virus (anti-HCV Ab).Fasting lipid profile (cholesterol levels, triglyceride, HDL, LDL). Fasting blood glucose level, fasting blood insulin level and calculation of insulin resistance using HOMA IR (patients were considered to have insulin resistance when HOMA IR > 2.5 (Nakai et al., 2002). Fasting serum Adiponectin level was measured using AviBion human adiponectin ELISA kit.Imaging workup including: -Abdominal ultrasound. Contrast Triphasic spiral abdominal CT as a part of the work-up in HCC diagnosis of the group of HCC.

*Exclusion criteria of our study*

*- Diabetes mellitus*

- Patients with other liver diseases (NAFLDs, autoimmune hepatitis, Wilson’s disease, alpha 1 antitrypsin deficiency, primary biliary cirrhosis, primary sclerosing cholangitis, alcoholic liver disease, drug induced hepatitis)

- Patients who received either forms of HCC treatment (Resection, Radiofrequency ablation, trans-arterial chemoembolization, radioembolization and systemic targeted agent like sorafenib).

- Current or past history of alcohol intake.

- Current or past history of any other malignancy.

- Patients on lipid lowering drugs and/or steatosis inducing drugs (corticosteroids, amiodarone, tamoxifen and valproic acid).

*Statistical analysis*

The collected data were tabulated and statistically analyzed using SPSS software (Statistical Package for the Social Sciences, version 16, SPSS Inc. Chicago, IL, USA). For quantitative data, the range, mean and standard deviation were calculated. For qualitative data, the comparison between two groups was done by Chi-square test. For comparison between more than two means of data, F value of ANOVA test was calculated. Pearson correlation coefficient (r) test was used to express the change between two different variables with each other in a linear fashion. Significance was at p ≤ 0.05 for the results of tests of significance.

## Results

The study included 100 patients who were divided into: Group I: 52 HCV patients with chronic liver disease (CLD), they were 32 (61.5%) men and 20 (38.5%) women, their mean age: 52.5±6.8 years and their mean BMI: 27.7±2.0 Kg/m^2^. Group II: 48 patients with hepatocellular carcinoma (HCC), they were 26 (54.2%) men and 22 (45.8%) women, their mean age: 53.2±7 years and their mean BMI: 25.2±1 Kg/m^2 ^(Table 1).

The study showed that sex and age of the patients of the two studied groups were not statistically different .BMI was significantly higher in group I (27.736±2.026 Kg/m^2^) than in group II (25.203±0.937 Kg/m^2^) (p=0.000) as shown in Table 1.

According to the liver profile, the ALT and alpha fetoprotein (αFP) were significantly higher in group II than group I (p=0.0001). The lipid profile showed that total cholesterol and triglycerides (TG) were significantly higher in group I than in group II (p=0.009 and p=0.003 respectively); LDL-C was significantly higher in group II than in group I (p=0.001), while HDL-C showed no significant difference between the two groups. As regard mean values of fasting glucose, insulin and HOMA index of insulin resistance, they showed higher significant values in group II (p=0.0001). Moreover, the study showed that serum adiponectin level in group II was significantly lower than in group I (p=0.0001) (Table 2).

Regarding Child Score of our studied patients, score (A) was only found in 12 patients in group I (p=0.000), Child score (B) was 19 patients in group I and 27 in group II representing (36,538% and 56.25% respectively) (p=0.238), Child score (c) was 21 patients in both group I and II representing (40.384% and 43, 75% respectively) (p=1) (Table 5).

It was found that mean values of Adiponectin were inversely correlated to age, α fetoprotein levels (p=0.0001) and child score (p=0.01) in Group I , but not in Group II .on the other hand, mean values of Adiponectin were inversely correlated to BMI among Group I (p=0.02) and Group II (p=0.11). Mean values of Adiponectin were inversely correlated to number and overall size of HFL in HCC group (p=0.01). There is no significant correlation of adiponectin level to insulin resistance parameters (fasting serum insulin levels, fasting blood glucose levels, and HOMA-IR index) as shown in Table 3. There is no significant correlation of insulin resistance to studied variables (Table 4).

Roc curve analysis for serum Adiponectin revealed that cut off value of 2.4μg/ml can discriminate HCC from chronic HCV cases with sensitivity 97.9% and specificity 100%. AUC= 0.0001 with 95% CI making the p value 0.0001 which is very highly significant.

Roc curve analysis for serum Insulin resistance revealed that cut off value of 2.20 can discriminate HCC from chronic HCV cases with sensitivity 100% and specificity 100%. AUC= 1.25with 95% CI making the p-value 0.0001 which is very highly significant.

**Table 1:** Demographic and Clinical Findings of the Studied Groups

Parameters		Group I	Group II	P
		CLD	HCC	
Sex n (%)	Male N=58	32 (61.53%)	26 (54.16%)	0.455
	Female N=42	20 (38.46%)	22 (45.83%)	
Age (years)	Range	36-63	39-65	0.618
	Mean±S.D.	52.538±6.812	53.229±6.981	
BMI (Kg/m^2^)	Range	24-31.12	22.97-27.21	0.000
	Mean±S.D	27.736±2.026	25.203±0.937	

CLD, chronic liver disease; HCC, hepatocellular carcinoma; BMI, body mass index

**Table 2:** Biochemical Results of the Studied Groups

Parameters		Group I	Group II	P
		CLD	HCC	
ALT (mg/dl)	Range	42-120	63-123	0.0001
	Mean±S.D	76.096±20.335	88.916±12.97	
αFP	Range	10-88.1	410-15482	0.0001
	Mean±S.D	42.413±21.945	4556.166±4613.49	
T. Cholesterol (mg/dl)	Range	150-181	153-171	0.009
	Mean±S.D	166.44±7.73	162.916±4.980	
TG3 (mg/dl)	Range	75-131	77-114	0.003
	Mean±S.D	104.711±16.522	96.104±10.314	
HDL-C (mg/dl)	Range	36-50	35-51	0.098
	Mean±S.D	42.346±4.048	43.708±4.099	
LDL-C (mg/dl)	Range	80-115	85-126	0.001
	Mean±S.D	94.923±9.469	102.104±11.567	
Fasting glucose (mg/dl)	Range	70-101	80-103	0.000
	Mean±S.D	81.0104±7.565	89.711±6.209	
Fasting insulin (µu/ml)	Range	17-27.20	21.12-28.40	0.000
	Mean±S.D	21.387±2.267	25.2±1.94	
HOMA IR index	Range	3.20-6.5	4.20-8.80	0.000
	Mean±S.D	5.183±0.910	6.892±1.161	
Adiponectin (μg/ml)	Range	9.5-16.4	4.8-9	0.0001
	Mean±S.D	12.365±2.166	6.731±1.244	

CLD, chronic liver disease; HCC, hepatocellular carcinoma; ALT, alanine aminotransferase; αFP, alpha fetoprotein; TG, triglycerides; HDL-C, high density lipoprotein-cholesterol; LDL-C, low density lipoprotein-cholesterol; HOMA-IR, homeostasis model assessment; IR, insulin resistance.

**Figure 1:**
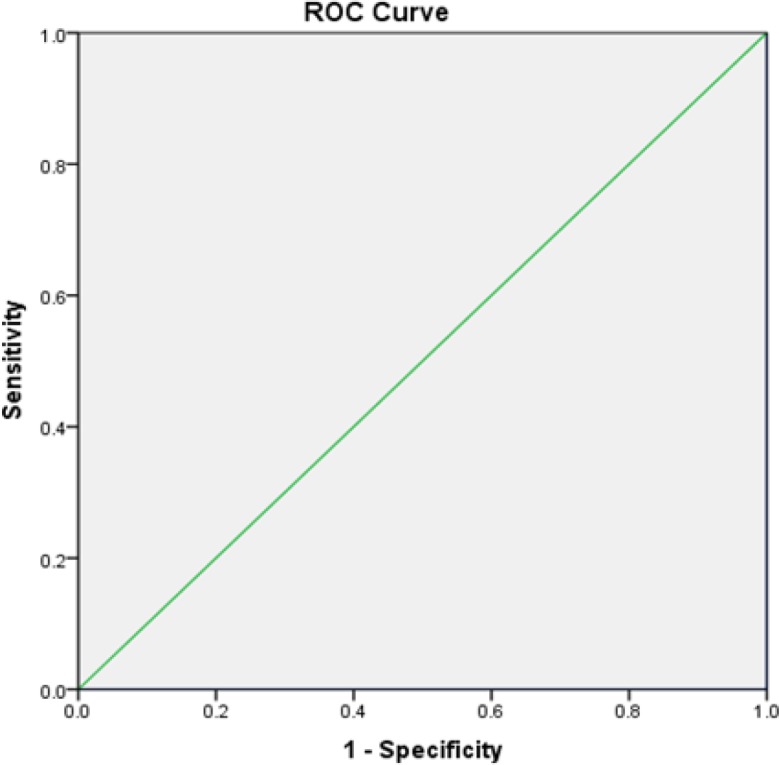
Roc Curve of Serum Adiponectin

**Figure 2:**
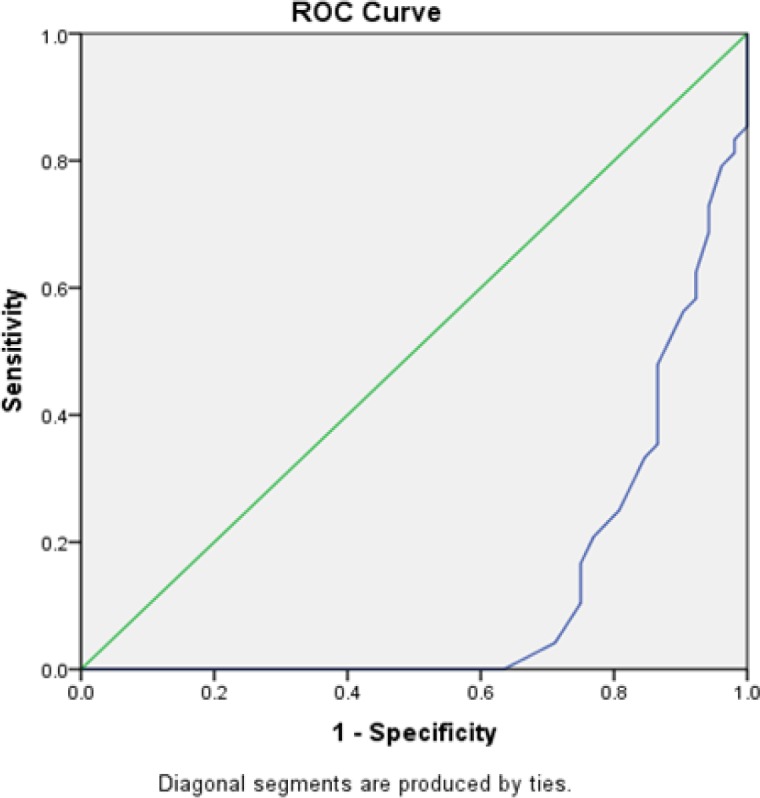
Roc Curve of Serum HOMA Insulin Resistance

**Table 3:** Correlation of Different Variables to Serum Adiponectin Level

Variables	Group I		Group II	
	R	P	R	P
Age	-0.828	0.0001**	-0.2005	0.1718
αFP	-0.659	0.0001**	-0.0824	0.5776
n. HFL			-0.7296	0.01*
Overall size of HFL			-0.8625	0.01*
Child score	-0.891	0.01*	0.06412	0.6651
BMI	-0.321	0.02*	-0.364	0.011*
Fasting glucose (mg/dl)	-0.039	0.784	0.107	0.467
Fasting insulin (µu/ml)	0.026	0.857	-0.172	0.242
HOMA IR index	-0.205	0.144	-0.010	0.949

αFP, alphafetoprotein; HFL, Hepatic Focal Lesion n. HFL, number of Hepatic Focal Lesion; BMI, Body Mass Index. *, p value is significant. **, p value is highly significant.

**Table 4:** Correlation of the Studied Variables to Insulin Resistance

Variables	Group I		Group II	
	R	P	R	P
Age	0.1624	0.250	-0.1041	0.481
αFP	0.34149	0.0132	0.0632	0.669
n. HFL			-0.0460	0.756
Child score	0.2800	0.0443	0.1130	0.444
BMI	0.1892	0.1790	-0.0607	0.6819

αFP, alphafetoprotein; HFL, Hepatic Focal Lesion n. HFL, number of Hepatic Focal Lesion; BMI, Body Mass Index.

**Table 5:** The CHILD Score of the Two Studied Groups

Child score		Group I	Group II	Total	Chi square					
					X²	P	X²	P	X²	P
A	(n)	12	0	12	12	0.000				
	%	23.07%	0.0%							
B	(n)	19	27	46	1.39	0.238	0.67	0.413	13.253	0.001
	%	36.538%	56.25%							
C	(n)	21	21	42	0	1				
	%	40.384%	43.75%							

**Table 6:** Roc Curve Analysis for Adiponectin and Insulin Resistance

Variables	Best cutoff	Sensitivity (%)	Specificity (%)	Positive predictive value (%)	Negative predictive value(%)
Adiponectin μg/ml	2.4	97.9	100.00	100.00	98.00
Insulin resistance	2.20	100.00	100.00	100.00	100.00

## Discussion

Hepatocellular carcinoma (HCC) has become the fastest rising cause of cancer-related deaths (El Serag, 2011). HCC prognosis is poor due to frequent intrahepatic spread and extrahepatic metastasis (Lafaro et al., 2015). The majority of HCC patients have no effective treatment due to advanced disease at the time of diagnosis (Njei et al., 2015).

Adiponectin is the most abundant adipokine which circulates at the highest levels in human plasma and synthesized by adipose tissue and has been shown to be a key component in the relationship between adiposity, insulin resistance and inflammation (Silva et al., 2014). Evidence suggests an association between adiponectin and liver tumorigenesis (Sharma et al., 2010).

Obesity and metabolic syndrome are recognized risk factors for hepatic steatosis (Monto et al., 2002), severe fibrosis (Jonsson et al., 2006), and HCC in patients with chronic hepatitis C (CHC) (Ioannou et al., 2007; Ohki et al., 2008). 

Chronic liver diseases are commonly associated with insulin resistance (Bugianesi et al., 2005). In addition; serum insulin levels were higher in diabetic patients with chronic liver disease, than those patients with lifestyle-type 2 DM (Kawaguchi and Sata, 2010).

Beside the common factors such as obesity and sedentary lifestyle, the development of insulin resistance may be due to several pathogenic factors that may trigger the pathophysiology of hyperinsulinemia in patients with chronic liver disease, (Kawaguchi and Sata, 2010) which include hepatic parenchymal cell damage, portal-systemic shunting and hepatitis C virus (Kawaguchi et al., 2004).

The current study showed insignificant difference of age and gender of our studied groups. These findings came in discordance with Hung et al., (2010). 

On the other hand our study showed agreement with Hung et al., (2010) study done in 2010, in that there is a significant difference between patients with CLD and patients with HCC as regard lipid profile and all IR parameters (fasting serum insulin, fasting blood glucose and HOMA-IR). This finding is in accordance with Khattab et al., (2012) as their study showed that the HCC group showed significantly higher levels of insulin, glucose, HOMA-IR as well as lower levels of total cholesteroland triglycerides compared with the matched CHC patients. And in harmony with Sadik et al., (2012) who found a significant increase in serum glucose level in all HCC patients as compared with patients with only liver cirrhosis and normal healthy controls. Hyperglycemia was reported to promote tumorgenesis by several pathways. The elevated level of serum glucose is advantageous for the increased DNA synthesis of the tumor cells. It provokes release of free radicals which will cause derangement of both the DNA and the enzymes having a role in the repair mechanisms (Suba and Ujpa´l, 2006). However, this relation is controversial as some researchers found it insignificant (Craik et al., 2011; Hamdy et al., 2015).

Considering the serum adiponectin level, the current study showed a highly statistically significant difference(p=0.000) between patients with CLD and patients with HCC, which was lower in HCC patients than CLD patients, these results is in agreement with Saxena et al., (2010), they concluded that adiponectin has a protective effect against hepatic carcinogenesis. Also, decreased circulating adiponectin level may play a role in the development of HCC and poor prognosis specially in obese HCC patients, Shams et al., (2011), found that the mean serum adiponectin level was significantly lower in HCC cases whom concluded that decreased circulating adiponectin level may play a role in the development of HCC and is a potential poor prognostic marker. Hamdy et al., (2015) also found highly significant lower levels of adiponectin in cirrhotic patients with HCC than in cirrhotic patients without HCC.

On the contrary, Arano et al., (2010), Chen et al., (2012) and Khattab et al., (2012) revealed the opposite results; it may be due to different race, ethnicity and/or number of patients.

When a correlation comparison was done between different variables with serum adiponectin level, it revealed highly negative significant correlation with BMI in both groups; this was in agreement with Chen et al., (2012) whose correlation analysis showed an inverse correlation between serum adiponectin levels and BMI in the HCC patients and control subjects. 

While in the current study correlations between IR parameters (fasting serum insulin levels, fasting blood glucose levels, and HOMA-IR index) and Adiponectin in both groups; was not significant.

In comparison, Chen et al., (2012), found that serum adiponectin levels was inversely significantly correlated with insulin resistance in the controls, this was not observed in the HCC patients.

In addition, Hamdy et al., (2015), revealed highly significant negative correlations between serum adiponectin levels and all parameters of IR (fasting serum insulin levels, fasting blood glucose levels, and HOMA-IR index) in all cirrhotic patients (patients with and without HCC).

Serum adiponectin level and both the number of hepatic Focal lesions and the overall size of the tumors showed significant negative correlations were also reported by (Khattab et al., 2012; Hamdy et al., 2015). This interesting finding may suggest an intimate relationship between metabolic disorder and HCV-related HCC (Khattab et al., 2012).

Regarding the cut off value of serum adiponectin and serum insulin resistance were used to analyze the effectiveness of those tests (If area under the curve [AUC] is near 1 it has higher chance of correct classification as in serum Insulin resistance test. However, if the AUC is near 0, higher chance of incorrectly classifying in opposite group. But when the value is 0.5; the test is no better than just tossing a coin for classification into positive or negative). In addition, the high sensitivity results in low number of false negative cases while high specificity leads to low number of false positive cases. Therefore, depending on the situation, requirement and seriousness of loss due to misclassification optimal value of sensitivity and specificity is decided and the test value corresponding to this may be taken as cutoff value for classification.

In conclusion, taken together, based on the findings of our study, it can be concluded that Insulin Resistance is highly associated with the development of HCC. 

The relation of serum adiponectin levels and presence of cirrhosis and/or HCC shed a light on that marker to be used in the future for prognostic and therapeutic implications in CLD patients.

The limitation of that study, the limited number of patients, the ignorance of the degree of fibrosis, HCV loads by PCR. They may have a significant impact. 

*Recommendation*

Further examination of the potential association between adiponectin, HOMA IR and liver cancer risk is warranted on a large scale.

Assess and evaluate the role of adiponectin in prognosis and prediction of survival to HCC patients.
